# Accurate RNA Sequencing From Formalin-Fixed Cancer Tissue to Represent High-Quality Transcriptome From Frozen Tissue

**DOI:** 10.1200/PO.17.00091

**Published:** 2018-01-26

**Authors:** Jialu Li, Chunxiao Fu, Terence P. Speed, Wenyi Wang, W. Fraser Symmans

**Affiliations:** **Jialu Li**, **Chunxiao Fu**, **Wenyi Wang**, and **W. Fraser Symmans**, The University of Texas MD Anderson Cancer Center, Houston, TX; and **Terence P. Speed**, University of California, Berkeley, Berkeley, CA; and Walter and Eliza Hall Institute of Medical Research, Parkville, Australia.

## Abstract

**Purpose:**

Accurate transcriptional sequencing (RNA-seq) from formalin-fixed paraffin-embedded (FFPE) tumor samples presents an important challenge for translational research and diagnostic development. In addition, there are now several different protocols to prepare a sequencing library from total RNA. We evaluated the accuracy of RNA-seq data generated from FFPE samples in terms of expression profiling.

**Methods:**

We designed a biospecimen study to directly compare gene expression results from different protocols to prepare libraries for RNA-seq from human breast cancer tissues, with randomization to fresh frozen (FF) or FFPE conditions. The protocols were compared using multiple computational methods to assess alignment of reads to a reference genome, the uniformity and continuity of coverage, the variance and correlation of overall gene expression, patterns of measuring coding sequence, phenotypic patterns of gene expression, and measurements from representative multigene signatures.

**Results:**

The principal determinant of variance in gene expression was use of exon capture probes, followed by the conditions of preservation (FF *v* FFPE) and phenotypic differences between breast cancers. One protocol, with RNase H-based ribosomal RNA depletion, exhibited the least variability of gene expression measurements and strongest correlation between FF and FFPE samples and was generally representative of the transcriptome from standard FF RNA-seq protocols.

**Conclusion:**

Method of RNA-seq library preparation from FFPE samples had a marked effect on the accuracy of gene expression measurement compared with matched FF samples. Nevertheless, some protocols produced highly concordant expression data from FFPE RNA-seq data, compared with RNA-seq results from matched frozen samples.

## INTRODUCTION

Although it is generally best to identify gene expression biomarkers from cancer tissues using the highest quality of RNA purified from fresh frozen (FF) samples, any subsequent development toward diagnostic testing will require translation for use with formalin-fixed paraffin-embedded (FFPE) tissue samples. However, the variably fragmented and chemically modified RNA derived from FFPE samples presents a challenge for accurate measurement of gene expression.^1,2^

In a different context, there is great interest to perform transcriptome sequencing (RNA-seq) for biomarker discovery research using large cohorts of precious archival FFPE samples from completed clinical trials. However, an unfavorable signal-to-noise ratio from FFPE samples could reduce the accuracy of biomarker discovery. Therefore, it is essential to select a protocol for FFPE RNA-seq libraries that yields data that are comparable with a gold standard result from FF samples. But there is more than one standard protocol for RNA-seq of high-quality RNA from FF tumor samples. Different approaches to generating libraries for RNA-seq include selection of mRNA by targeting the poly(A) tail (mRNA protocol), depletion of more abundant ribosomal RNA (rRNA depletion) using bead-based method (I.TotalRNA protocol) or enzymic method (K.TotalRNA protocol), and exon capture probes for known coding region (CR) sequence from an RNA-seq library prepared (CR protocol).

Data generated from the popular mRNA protocol using FF tissue samples (FF.mRNA library) are highly concordant with microarray data in tumor gene expression signature studies.^3^ This protocol is not appropriate for degraded mRNAs from FFPE samples, however.^4^ On the other hand, total RNA library protocols do not restrict enrichment to poly(A)^+^ tailed mRNA, allowing less biased quantification of isoform abundance.^4-6^

**Fig 1.**
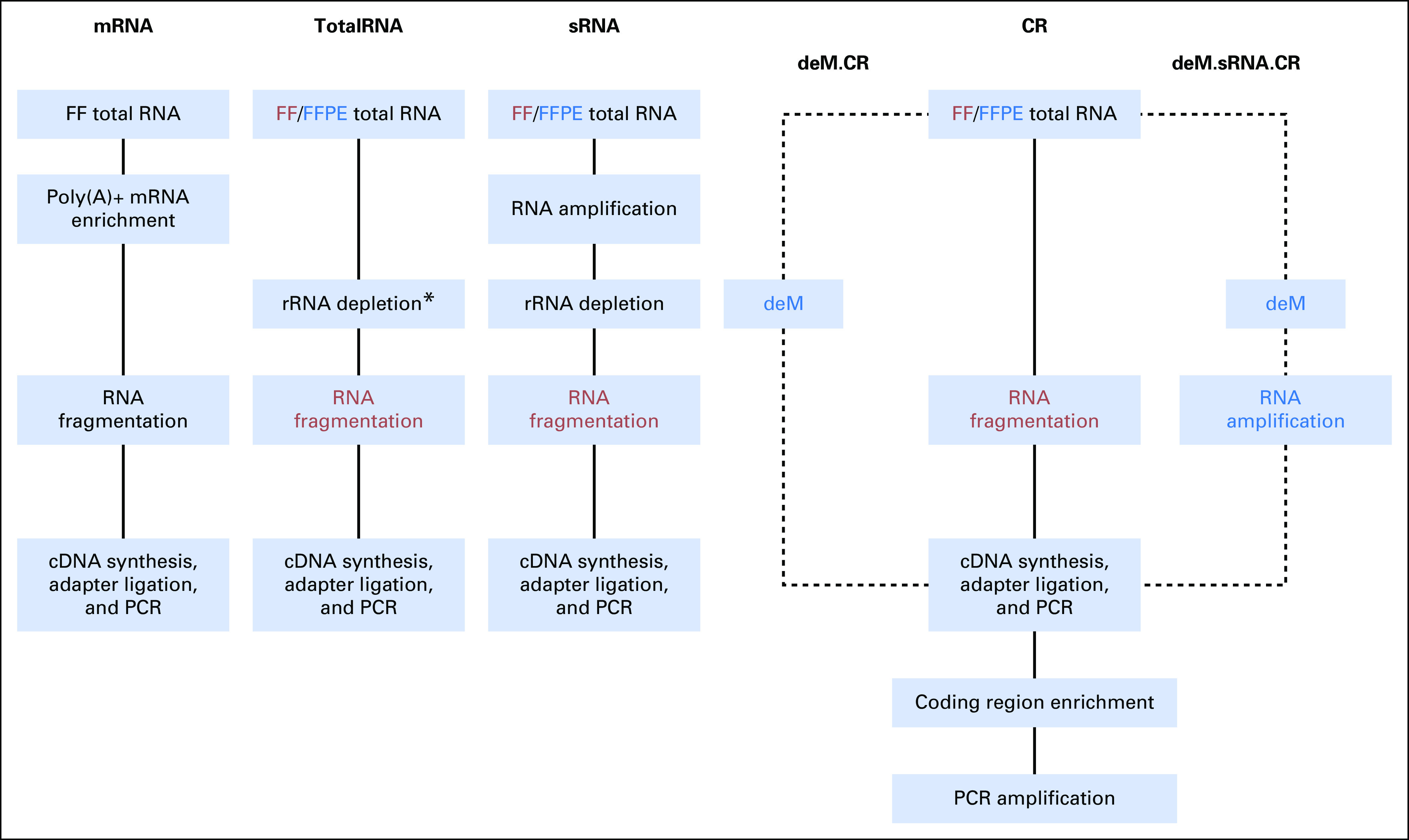
Workflows of RNA-seq library preparation. Red indicates steps only applied to fresh frozen (FF) samples, and blue indicates steps only applied to formalin-fixed paraffin-embedded (FFPE) samples. (*) Indicates different ribosomal RNA (rRNA) depletion methods that result in two different TotalRNA protocols, that is, RiboZero for I.TotalRNA and RNase H for K.TotalRNA protocol. CR, coding region capture protocol; deM, demodification protocol; mRNA, mRNA-targeting protocol; PCR, polymerase chain reaction; sRNA, sense RNA protocol.

Corresponding protocols for RNA-seq from FFPE tumor samples include an adaptation of the mRNA protocol that combines random and poly(A) primers (sense RNA [sRNA] protocol) optimized for gene expression microarrays (SensationPlus kit; Affymetrix, Santa Clara, CA) or are unchanged for the I.TotalRNA, K.TotalRNA, and CR protocols (Fig 1). Total RNA protocols have achieved Pearson correlations with FF counterparts of > 0.9.^4,6,7^ Exon capture using the CR protocol has potential for stronger correlation but involves selected coverage.^8^ Finally, because pretreatment heat and methyl saturation are claimed to reduce methylol adducts on FFPE RNA,^9^ we evaluated preanalytical demethylation of total RNA before library preparation using the CR and sRNA protocols (Fig 1).

Consequently, this study was designed to directly compare the results from RNA-seq library protocols between optimally matched sample pairs (FF and FFPE) from representative breast cancers, to address three scenarios in translational research: biomarker discovery from FF samples phase, with intention to translate for FFPE samples in future studies for validation and diagnostic development; biomarker discovery from FFPE samples that is intended to be representative had high-quality FF samples been available; and translation of existing biomarkers, developed using a different method (such as microarrays or RNA-seq using mRNA protocol), for use with RNA-seq data from FFPE samples.

## METHODS

### Tumor Tissue Samples

Fresh tumor tissue was collected at intraoperative pathology evaluation, diced into pieces of 1- to 2-mm diameter, stirred, and randomly assigned to RNAlater solution later stored in a −80°C freezer (FF) or 10% neutral buffered formalin and paraffin-embedded as an FFPE tissue block.^10^ Phenotypically, the nine breast cancers were defined by the pathologic status of hormone receptors (HRs) and human epidermal growth factor receptor 2 (HER2) as HR-positive/HER2-negative in five, HR-positive/HER2-positive in one, and triple receptor-negative in three. RNA was purified from FF samples using the RNeasy Mini Kit (Qiagen, Valencia, CA) and FFPE samples from 10-μm sections using High Pure FFPE RNA Isolation Kit (Roche, Indianapolis, IN). A DNase-I treatment step was included in both.

### Construction of RNA-seq Library and Sequencing

Full details of all methods for library construction and sequencing of RNA samples are in the Data Supplement. An overview diagram of the different RNA-seq library protocols is shown in Figure 1, and details of the number of libraries prepared, starting RNA requirement, cost, and duration to perform each protocol are summarized in the Data Supplement.

The mRNA protocol (FF only) used oligo-dT beads for poly(A)^+^ mRNA enrichment, followed by standard procedures of TruSeq RNA Sample Prep Kit v2 (Illumina, San Diego, CA). The I.TotalRNA protocol used Ribo-Zero Magnetic Gold Kit to deplete ribosomal RNA (rRNA) from total RNA, followed by library preparation using the Truseq Stranded Total RNA Sample Prep Kit (Illumina). The K.TotalRNA protocol used an RNase H-based method to deplete rRNA from total RNA, followed by library preparation using KAPA Stranded RNA-Seq Kit with RiboErase (Kapa Biosystems, Wilmington, MA). The sRNA protocol used SensationPlus Amplification kit (Affymetrix) with oligo-dT and random primers designed for whole-transcriptome amplification. The sRNA was then synthesized by in vitro transcription, followed by rRNA depletion using the Ribo-Zero Magnetic Gold Kit. Then the rRNA-depleted sRNA was used as the template for the mRNA protocol described above.

The CR protocol was performed using Truseq Access RNAseq kit (Illumina), using random primers. Next, sequencing adapters were ligated to the resulting cDNA followed by the first polymerase chain reaction (PCR; 15 cycles). The CRs in those libraries were enriched using capture probes and amplified by PCR.

The demodification (deM) protocol used heat in an amine-rich solution (70°C for 30 minutes in 1 × Tris-EDTA buffer containing 20 µM NH_4_Cl, pH7.0).^9,11^ Starting with demodified RNA, we tested two additional FFPE library preparation methods: FFPE.deM.CR and FFPE.deM.sRNA.CR.

Libraries were randomly assigned to a lane (four per lane) and paired-end sequenced with Illumina HiSeq 2000 Sequencing System. We generated 100 base-paired reads for sample C and 50 base-paired reads for the other eight samples for the FF.mRNA and FFPE.sRNA protocols. All remaining libraries had 75 base-paired reads. For the mRNA and sRNA protocols, the libraries were prepared with two technical replicates to test reproducibility. No technical replicates could share the same sequencing lane.

### RNA-seq Data Analysis

Full details of all data analysis methods and an overview diagram of the analysis plan are provided in the Data Supplement. Briefly, the different protocols for FF and FFPE samples were compared with respect to metrics as follows: mapping rates of RNA-seq reads (exonic, intronic, intergenic), read coverage uniformity and continuity, principal component analysis and hierarchical clustering analysis on expression levels, and pairwise comparison per gene over the coding sequence, of all genes, and of selected breast cancer gene expression signatures that had previously been developed from reverse transcription–PCR or microarray data from FF samples.

## RESULTS

RNA extracted from FFPE samples was severely degraded, with RNA integrity number of 1.2 to 2.2, versus 6.7 to 9.3 from FF samples (Data Supplement). All libraries generated > 49 million raw reads (mean, 113 million; SD, 27 million).

### Postalignment Statistics

Postalignment mapping rates from FFPE samples differed from FF samples when using libraries from mRNA and TotalRNA protocols as follows: fewer exonic (overall mean difference, 0.335; *P* < .001), more intronic (overall mean difference, 0.309; *P* < .001), and comparable for intergenic sequence reads (Data Supplement). The RNAseq data generated from the sRNA protocol had a significantly lower concordant pair alignment rate as compared with those from non-sRNA protocols (*P* < .001; Data Supplement). Using the CR protocol, mapping was highly concordant between FF and FFPE and the exonic mapping rate was increased compared with non-CR methods (Data Supplement). Overall, the number of genes with read coverage (transcripts per million [TPM] > 0.1) was slightly higher in FFPE samples than in FF samples for both non-CR and CR protocols (Data Supplement).^12^

### Uniformity and Continuity of Read Coverage of Transcripts

Uniformity of read coverage was measured by the mean coefficient of variation, and continuity of coverage as the percentage of gaps without read coverage, across the top 1,000 highly expressed transcripts (Data Supplement). FFPE.I.TotalRNA and FFPE.K.TotalRNA libraries demonstrated the most uniform and continuous coverage among protocols for FFPE samples and were equivalent to protocols for FF samples. In contrast, the CR protocol produced nonuniform coverage, with a high percentage of gaps, in both FF and FFPE libraries. The FFPE.sRNA protocol introduced modest nonuniformity.

### Preanalytical Sources of Variance

**Fig 2.**
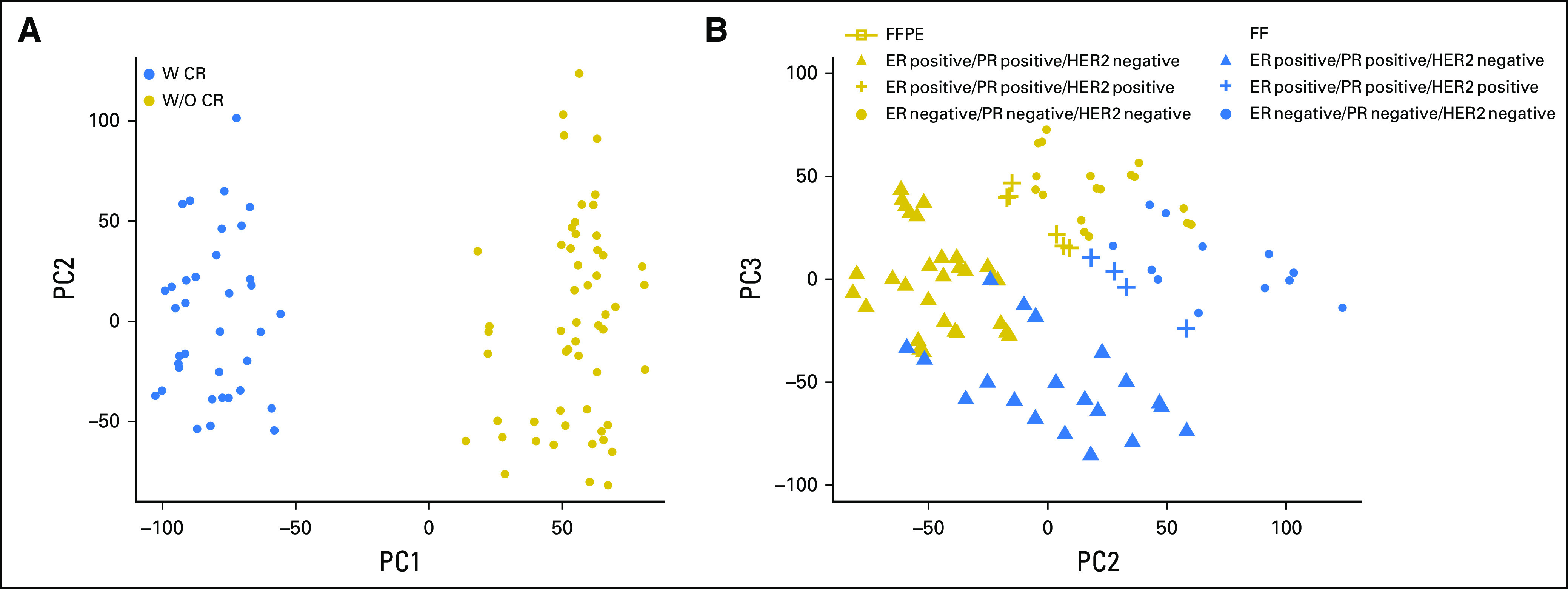
Scatter plot of the first three principal components for count per million–normalized and variance stabilizing transformed counts of 20,381 coding region (CR)-targeted poly(A)^+^ genes. Each point corresponds to one of 90 libraries. (A) Blue circles indicate samples prepared with CR and gold circles indicate those without CR treatment; 26.5% of total variation comes from CR treatment. (B) Blue color indicates fresh frozen (FF) samples and gold for formalin-fixed paraffin-embedded (FFPE) samples. The symbol shape indicates the different biologic group. The biologic differences and FFPE effects are captured, which accounts for 20.6% of total variation. ER, estrogen receptor; HER2, human epidermal growth factor receptor 2; PC, principal component; PR, progesterone receptor; W CR, with coding region capture; W/O CR, without coding region capture.

In RNA-seq studies, the variance across samples usually grows with the mean of gene expression (also known as heteroscedasticity), and this can be problematic for correctly uncovering the underlying pattern in data using techniques such as distance-based clustering.^13^ We therefore applied the variance-stabilizing transformation method to approximate the independence between variance and mean (Data Supplement). Principal component analysis of expression of a total of 20,381 CR protocol targeted poly(A)^+^ genes for all libraries showed that the 26.5% of total variation captured by the first principal component was due to use of exon capture probes (CR protocol), and 20.6% from the second and third components due to the combined effects of FFPE and biologic differences (Fig 2). Hierarchical clustering results, with high confidence (average bootstrap probability, 0.93), showed that the major tumor phenotypes (HR-positive *v* HR-negative) and the source tumor clustered together with FFPE samples (Data Supplement). Technical replicates (both FF.mRNA and FFPE.sRNA protocols in all nine tumors) were highly correlated (Spearman ρ ≥ 0.992) for all samples after normalization by total count and transformation to log_2_ count per million (Data Supplement).

### Protocols That Target mRNA or Deplete rRNA

**Fig 3.**
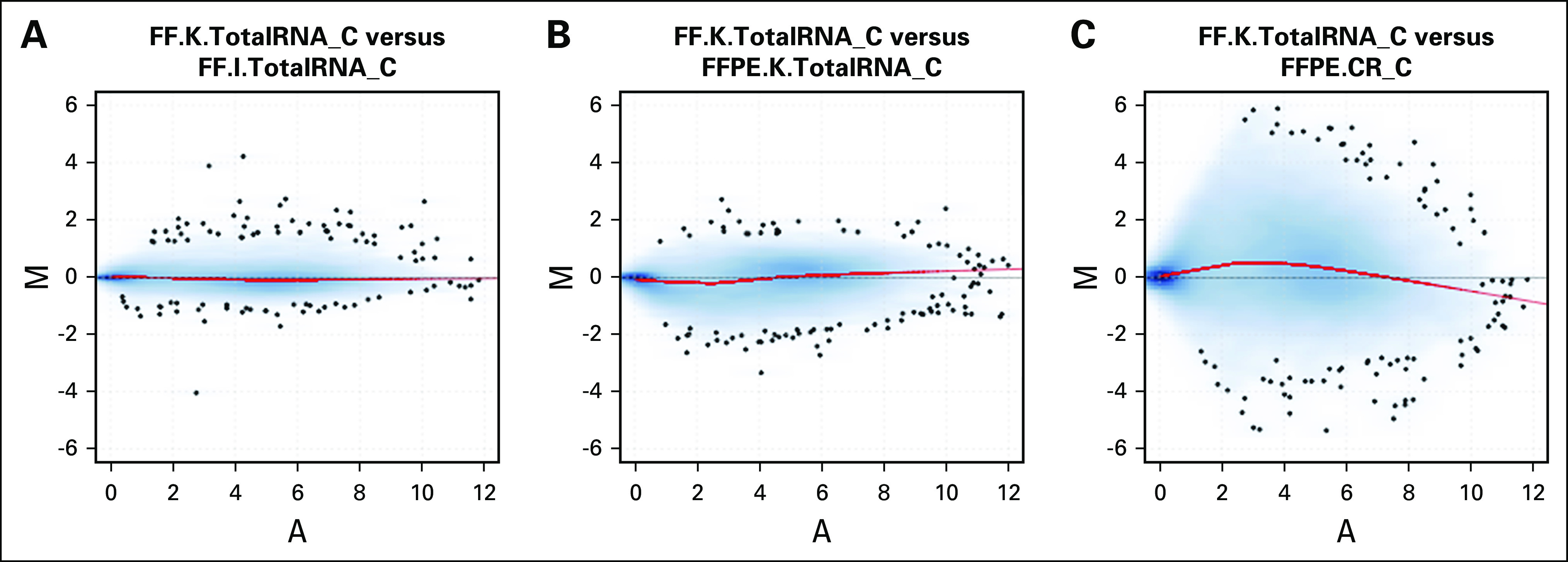
Difference in measurements as log ratio (M) versus mean average expression (A) of each gene (MA plot) of 20,381 coding region (CR)-targeted poly(A)^+^ genes for tumor sample C when using FF.K.TotalRNA sample C library as the reference. (A) MA plot for tumor C between FF.K.TotalRNA and FF.I.TotalRNA; (B) MA plot for tumor C between FF.K.TotalRNA and FFPE.K.TotalRNA; (C) MA plot for tumor C between FF.K.TotalRNA and FFPE.CR. M is the log_2_-transformed expression of a gene from first library divided by that from the second library, and A is the mean log_2_-transformed expression of the gene. The red curve indicates the Lowess smoother fitted to the data. FF, fresh frozen; FFPE, formalin-fixed paraffin-embedded.

Figure 3 illustrates, for one tumor (C), MA plots of gene expression for pairs of libraries. Comparing both TotalRNA protocols with FF samples, differences were centered around zero, with small variation across different mean expression levels (Fig 3A). Comparing FF and FFPE samples using the same TotalRNA protocol, the log ratio values were still centered around zero at different mean expression levels (Fig 3B). However, comparing the FFPE.CR protocol to the FF reference, the log ratio values deviated from zero at both low and high expression levels (Fig 3C). The same patterns were observed for all other tumor samples (Data Supplement). These observations suggest that the TotalRNA protocols produced high-quality FFPE RNA-seq data that were comparable to the FF RNA-seq data.

**Fig 4.**
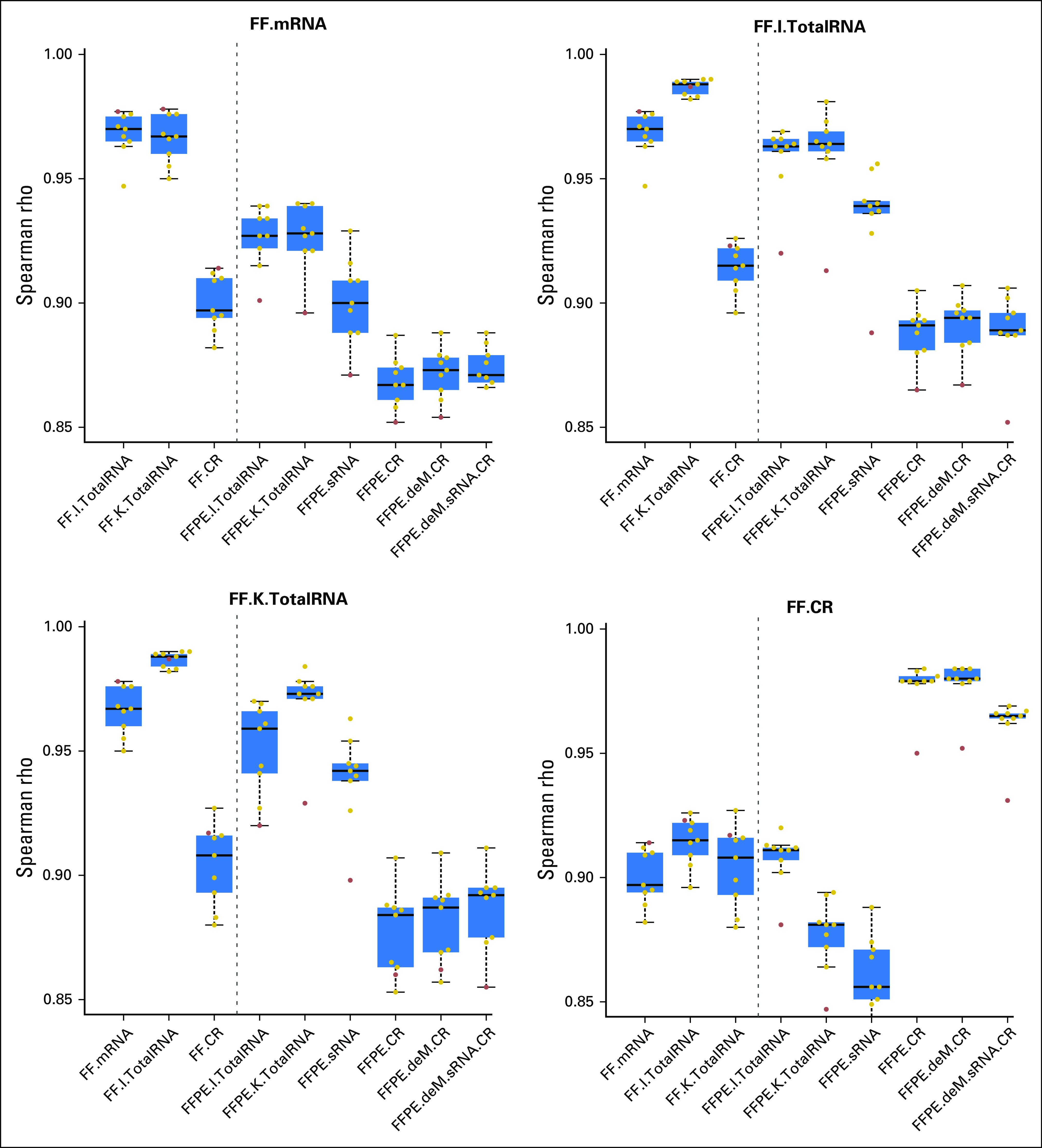
Summary of between-protocol correlation coefficients on the basis of transcripts per million. The title of each figure is the reference protocol used for comparison. Each dot is the Spearman rho estimate calculated between the reference library and the library showing on the *x*-axis. Each box summarizes the Spearman rho estimates from nine breast tumor samples. The red dot indicates the tumor sample N. CR, coding region protocol; deM, demodification; FF, fresh frozen; FFPE, formalin-fixed paraffin-embedded; sRNA, sense RNA protocol.

The FF.K.TotalRNA and FFPE.K.TotalRNA libraries had highly correlated TPM measures, with median rank correlation 0.973. This was significantly higher than for FF.K.TotalRNA with FF.CR (mean difference, 0.066; *P* < .001) or any other FFPE protocol (least mean difference, 0.019; *P* = .031; Fig 4). Results were similar using count per million and fragments per kilobase per million measures of gene expression (Data Supplement). The FFPE.K.TotalRNA also had the highest median rank correlation with FF.mRNA and FF.I.TotalRNA, despite normalization methods used (Fig 4; Data Supplement).

### Protocol With Subsequent Exon Capture

Subsequent exon capture (CR protocol) resulted in a median rank correlation of 0.980 between FF and FFPE, but the FF.CR had much lower correlation with non-CR libraries (least mean difference, 0.063; *P* < .001 using TPM; Fig 4; Data Supplement). Generally, the CR protocol tended to overly enrich the highly expressed genes and was more likely to not capture low-expressed genes (Fig 3C; Data Supplement). This was not improved by prior demodification of methylol adducts from FFPE tissue–derived RNA using heat and amines or the sRNA protocol (Fig 4; Data Supplement). Although both approaches seemed to slightly increase concordance of expression, neither was statistically significant.

After further investigating these protocol-induced biases, we calculated the number of genes that would be considered as differentially expressed or false positives compared with each reference FF standard protocol (Data Supplement). Fewer FP genes would suggest fewer artifacts introduced by a protocol. FFPE.K.TotalRNA RNA-seq data had the fewest genes with significantly differential expression at various *P* value thresholds and using different data normalization methods. In contrast, FF.CR was the most biased method, compared with FF.mRNA, with 84.2% of all genes significantly differentially expressed at an adjusted *P* value cutoff of .01.

### Pattern Dissimilarity in Measurement of Coding Sequence

We used a pattern dissimilarity score to measure the differences in expression patterns of coding DNA sequences between library protocols, allowing direct comparison of non-CR and CR protocols. A smaller value of the score indicates higher similarity between a protocol and an FF reference. The distributions of dissimilarity scores across all genes were similar within each protocol but varied across protocols (Data Supplement). FFPE.K.TotalRNA had the lowest mean dissimilarity score when using FF non-CR libraries as the reference (Data Supplement).

### Gene Expression Patterns Associated With Tumor Phenotype

We analyzed differential expression (DE) of genes comparing HR-positive/HER2-negative and triple receptor–negative breast cancers within each protocol. Overall, the normalized data were distributed around zero relative log expression and were clustered by tumor phenotypes in the first two principal components. The *P* value from DE analysis followed the ideal uniform distribution for non-DE genes, with a spike close to zero for the DE genes (Data Supplement). Receiver operating characteristic curves represented the sensitivity and specificity of the DE analyses using each FF reference as the gold standard. FFPE.K.TotalRNA achieved high and stable area under the curve (0.921 to 0.933) at different cutoffs set for each FF gold standard, even after the strongest DE genes in the gold standards had been filtered out (Data Supplement). The best agreement between FFPE protocols and each FF standard was as follows: FFPE.sRNA with FF.mRNA, FFPE.K.TotalRNA with both FF.I.TotalRNA and FF.K.TotalRNA, and FFPE.CR with FF.CR (Data Supplement).

### Representative Gene Signatures of Prognosis

We compared five published breast cancer gene expression signatures: recurrence score (Oncotype DX), PAM50, sensitivity to endocrine therapy index, mammaprint, and PI3-kinase index (PI3K).^14-19^ Those were compared between three FFPE protocols (I.TotalRNA, K.TotalRNA, and sRNA) and three FF protocols (mRNA, I.TotalRNA, and K.TotalRNA). Best correlations using FFPE protocols with FF.mRNA (range, 0.911 to 0.934) were not as strong as with FF.I.TotalRNA (range, 0.952 to 0.975) or FF.K.TotalRNA (range, 0.956 to 0.986) protocols (Data Supplement). The FFPE.K.TotalRNA protocol had the highest observed Spearman correlation coefficient in 13 of these 15 comparisons.

## DISCUSSION

Overall, FFPE RNA-seq data reliably captured transcriptional profiles and differences in tumor phenotype-based expression in breast cancer samples, but not quite as well as FF RNA-seq data. Principal component analyses demonstrated the following order of variables influencing gene expression measurements from RNA-sequencing: whether the library preparation protocol used exon capture for CR, whether the sample was from FF tissue or FFPE tissue, and the biologic phenotype of the breast cancer based on HRs and HER2 receptor status (Fig 2). Generally, we observed small differences in performance between non-CR protocols. However, even small differences can have important effects on large-scale genomic data for biomarker discovery, validation, or subsequent diagnostic development. Nevertheless, we identified one protocol, FFPE.K.TotalRNA, with consistently good transcript coverage uniformity and continuity, most concordant expression, and least differential expression when compared with the different non-CR protocols with fresh tissue. This protocol used RNase H-based rRNA depletion method and outperformed another similar TotalRNA-seq method, which used RiboZero to remove rRNA. It had a reasonable requirement of total RNA input (100 ng) for FFPE biopsy samples.

The first translational research scenario that we posed, in the Introduction section, considered the best pairing of protocols that would enable discovery using FF samples with intention to later translate for use with FFPE samples. Overall, we favor the K.TotalRNA as consistently best or close to best performance with FFPE protocols when compared with FF.mRNA, FF.I.TotalRNA, or FF.K.TotalRNA as reference FF protocols. This interpretation was supported by the quality of read coverage, pattern of coding sequence expression, and translation of overall or phenotype-related gene expression profiles and prognostic signatures.

The CR protocols yielded concordant results, but different from all other (non-CR) protocols. Therefore, a CR protocol used for discovery (FF) would preclude other protocols for later translation to FFPE samples (Figs 2 and 4). Also, changes to the population of exon capture probes within a commercial kit over time could be a potential risk to this approach.

The most generalizable results for discovery research from FFPE samples were obtained using the Total.RNA protocols without exon capture. Although similar, the FFPE.K.TotalRNA protocol produced slightly stronger results than the FFPE.I.TotalRNA protocol. For our second scenario, therefore, we prefer the K.TotalRNA protocol for best representation of the transcriptome in FFPE samples used for discovery research—aiming to represent the transcriptional information that FF samples would have provided.

Our third translational research scenario involves the translation of an existing gene expression signature that was previously developed using a different method (eg, microarray) or a particular RNA-seq protocol. Again, the FFPE.K.TotalRNA protocol had the best performance for total transcriptional profile, coding sequence, phenotypic discrimination, and specific gene expression signatures.

The formalin fixation process is known to cause cross-linkage between nucleic acids and proteins and monomethyl addition to the RNA bases.^2^ Although we tested a method of chemical demodification of total RNA, our results showed negligible effect and argue against the incorporation of this method for RNA-seq of FFPE samples (Fig 4). However, we did not test the performance of potential protocols combining demodification with sRNA alone or TotalRNA methods, because of limited tumor sample total RNAs. The inclusion of random and dT primers and the T7 promoter region (sRNA protocol) to simulate the FF.mRNA protocol produced good concordance overall but introduced a high number of nonconcordant mapped reads, nonuniformity, and discontinuity of read coverage across the transcriptome.

Limitations to our study include small sample size (although cancers were selected to represent biologic diversity); optimally short time to fixation of tissues and possibly, as a result, a modest degree of degradation of FFPE samples (DV200 ranges from 65% to 85%); optimal amount of input RNA used for non-CR protocols (at least 100 ng); and lack of generalizability (single-institution conditions of tissue processing). Also, the effects of long-term storage of FFPE samples could not be tested—but would be expected from a completed clinical trial. Also, several of the cases had prolonged storage of cut FFPE sections (at 4°C) until RNA purification. This could have compromised the FFPE library protocols for this comparison but can also be viewed as stress testing the FFPE-derived RNA. One tumor (sample N) seems to be compromised by unknown technical processing; our study conclusions, whenever involving this sample, are based on robust point estimates (eg, median estimates in Fig 4) across all samples to avoid being driven by its outlier effect. Notwithstanding these limitations, we believe that the results from this study will be helpful to translational researchers as they consider how to obtain accurate gene expression by applying RNA-seq methods to FFPE tumor samples.
